# Species differences in comorbid alcohol use disorder and major depressive disorder: A narrative review

**DOI:** 10.1111/acer.70015

**Published:** 2025-03-09

**Authors:** Garrett A. Winkler, Nicholas J. Grahame

**Affiliations:** ^1^ Department of Psychology Indiana University Indianapolis Indianapolis Indiana USA

**Keywords:** alcohol abstinence, antidepressants, comorbidity, depression, preclinical models

## Abstract

Alcohol use disorder (AUD) and major depressive disorder (MDD) are often comorbid, and it is estimated that between 15 % to 33% of people dependent on alcohol have an MDD diagnosis. Mood‐related symptoms are also common in humans during acute withdrawal, but by most accounts, symptoms abate after 2–4 weeks of alcohol abstinence. Preclinical studies, important for understanding the etiology and finding treatments for this comorbidity, also find depression‐like and anxiety‐like phenotypes in early abstinence along with protracted negative affect detectable past 2 weeks postcessation. In this narrative review, we focus on the translational divergence of AUD and MDD comorbidity with a focus on the time line mismatch between species in concurrent AUD + MDD and MDD following AUD. We also highlight the preclinical success and clinical failure of classic antidepressants for AUD and the relative absence of withdrawal and negative affect in high‐drinking selected lines of mice and rats. We suggest sources of these discrepancies, including discussion of relief/reward‐driven drinking subpopulations and future directions for the field.

## Graphical abstract

In this narrative review, we identify barriers to the translation of preclinical comorbidity models of alcohol use disorder and depressive disorders, including temporal and pharmacological discrepancies. In humans, depression is high during and immediately after the cessation of drinking and usually remits subsequently, while in animals it seems to worsen during abstinence without much evidence for recovery. We suggest probing preclinical models that more closely recapitulate the time course and symptomatology of anhedonia and negative affect seen in these patients.

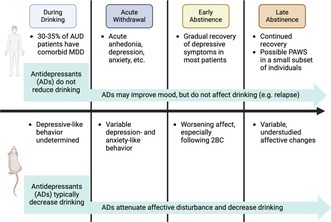

## INTRODUCTION

Major depressive disorder (MDD) and alcohol use disorder (AUD) are comorbid conditions; in fact, MDD is the most prevalent psychiatric disorder among individuals diagnosed with AUD using the definition from the *Diagnostic and Statistical Manual of Mental Disorders* (DSM‐5 (McHugh & Weiss, 2019)). Comorbidity worsens the prognosis of both disorders: AUD patients with comorbid depression have longer episodes of depressed mood and more frequent suicide attempts (Holma et al., 2020), respond less to common antidepressant treatments for MDD (Hashimoto et al., 2015), and have more frequent and intense bouts of drinking in the first month of AUD treatment compared with nondepressed controls (Conner et al., 2005).

These findings prompt debate over the directionality of causality between the two disorders. Clinical studies have revealed evidence for multiple pathways to comorbidity. In some cases, depression at an earlier timepoint predicts alcohol use disorders (Kuo et al., 2006; McCarty et al., 2009); in others, ongoing AUD increases risk for subsequent depressive episodes (Fergusson et al., 2009). Much of the longitudinal and prospective literature addresses adolescent depression or alcohol consumption predicting adult outcomes, and there is evidence for both pathways (Edwards et al., 2014; Pedrelli et al., 2016), but this review will focus on adult research only. MDD and AUD may interact causally or, like most human psychopathologies arise from shared genetic and environmental variables that predispose an individual toward one comorbidity trajectory or another (Dick, 2011; Underwood et al., 2018). For example, sex moderates the relationship between AUD and MDD—the lifetime prevalence of MDD in men and women with diagnosed alcohol dependence is 18.4% and 35.3%, respectively (Goldstein et al., 2012). Personality variables, including some personality traits recognized as important diagnostic criteria of personality disorders, also exert influence on the comorbidity relationship (Bornovalova et al., 2018). Suffice to say, the clinical picture of AUD–MDD comorbidity is complicated and multifactorial, spanning several theorized pathways, which hinge on developmental, genetic, and environmental variables.

Preclinical research can sometimes provide answers in situations where causality in clinical settings is complicated. In rodent models, genetic backgrounds can be simplified with inbred strain homozygosity or manipulated directly through selective breeding. In addition, environmental variables are more tightly controlled by the experimenter, at least theoretically. AUD–MDD comorbidity models attempt to capture different directions of the relationship. In a common MDD‐to‐AUD model, animals are given some stressful or frustrative challenge(s) in an attempt to model the human precipitation of depression via inescapable stress (van Praag, 2004; Willner et al., 1987). An increase in ethanol consumption in stressed versus unstressed controls may be interpreted as evidence for an MDD‐to‐AUD pathway. Here, chronic stress is more reliable (Anderson et al., 2016; Becker et al., 2011). The AUD‐to‐MDD model is the focus of this review. Here, animals are exposed to alcohol voluntarily or involuntarily; the alcohol is removed in “forced abstinence,” and days to weeks later, animals are screened for “negative affect,” which is defined as a collection of “anxiety‐like” and “depression‐like” behavioral phenotypes (Lezak et al., 2017; Slattery & Cryan, 2014). According to a recent mouse‐focused review from Bloch et al. (2022), a continuous (or two‐bottle choice, 2BC) drinking history provides the most replicability compared with other drinking paradigms, though even in the same strain, different laboratories have failed to replicate negative affect during forced abstinence using the same assays.

Across species, early abstinence may coincide with acute or protracted alcohol withdrawal states. For example, acute withdrawal in mice, measured via handling‐induced convulsions (HICs), is often observed in the 24 h following final exposure (Crabbe et al., 1980; Metten et al., 2010). In humans, early withdrawal can include not only convulsions in severe cases of AUD but also mood‐related complications resembling acute depression‐ and anxiety‐like states (Canver et al., 2024). Thus, it may be that more transient AUD‐associated withdrawal is being evaluated in these animal models rather than correlates of an MDD diagnosis in humans. That said, this period of early abstinence is when negative affect seems to be the most variable in mice (Bloch et al., 2022).

In this narrative review, we attempt to critique these models in hope of improving them, focusing on the discrepancy between humans and animals in the time course of negative affective symptomology during and after drinking. We also discuss the general failure of common antidepressants to lessen drinking in AUD despite strong preclinical promise from a range of studies. We then examine individual differences in depression and negative affect across rodent and human populations while suggesting sources of clinical vs. preclinical inconsistencies, including proposals for future directions of this AUD subfield and their potential confounds.

## AUD + MDD COMORBIDITY ACROSS SPECIES

In humans, depressive symptoms are often gauged using questionnaires and surveys, such as the Beck Depression Index (BDI) or Hamilton Depression Rating Scale (HDRS). Refer to Table 1 for brief descriptions of the measures that appear in this review.

**TABLE 1:** Clinical depression measures.

Clinical measures of depression	
Beck depression index (BDI)	A 21‐item (maximum) self‐rated questionnaire used to rate symptoms and thoughts/feelings common in patients with depression. Each item is rated 0–3, with 3 indicating the highest level of dysfunction, and the sum of the items indicates severity of depression. The BDI can be used to track the course of depression but is not a diagnostic tool
Hamilton Depression Rating Scale (HDRS)	A 17‐item interviewer‐rated questionnaire used to gauge depressive symptoms over the past week. Four additional questions may be added to identify depression subtype. The scale tends to focus on somatic or physical symptoms rather than thought patterns. The HDRS can be used longitudinally but is not a diagnostic tool
Mood and Anxiety Questionnaire (MASQ)	A 90‐item (maximum) self‐report Likert scale questionnaire using a tripartite model via questions about general distress or positive affect, depression‐specific symptoms, or anxiety‐specific symptoms. A higher score indicates more distress. The survey may be curtailed to investigate specific symptom clusters. The MASQ is not considered a diagnostic tool
Patient Health Questionnaire (PHQ‐2 & PHQ‐9)	The PHQ‐9 is a self‐report diagnostic tool for MDD consisting of nine questions about the frequency of common MDD symptoms over the past 2 weeks. The PHQ‐2 is just the first two items of the PHQ‐9 which measure anhedonia and dysphoria

In rodents, depression‐like behaviors are measured via behavioral tasks that attempt to capture aspects of human MDD. Approach, avoidance, and anxiety assays include tasks such as elevated plus/zero maze (EP/ZM), light/dark box (LDB), marble burying (MB), novelty‐suppressed feeding test (NSFT), and acoustic startle (AS). Anhedonia, despair, and stress coping assays include the forced swim test (FST), tail suspension test (TST), sucrose/saccharin preference test (SPT), and intracranial self‐stimulation (ICSS). Social interaction and irritability tests include social approach (SA) and social defeat (SD) (Bloch et al., 2022). For more details about these tasks, see Gencturk and Unal (2024).

### During drinking

Clinically, AUD and MDD often coexist. For example, in one study from Washington state including 300,000+ individuals, of those patients screening positive for depression (Patient Health Questionnaire [PHQ‐2] score ≥3), 5.2% were high‐risk drinkers, a 131% increase compared to depression‐negative patients. High‐risk drinking patients who screened positive for depression also had more incidence of diagnosed AUD (Ryan et al., 2022). Drinking level and pathological drinking thus can exert influence on concurrent depression severity, though this relationship seems to be nonlinear (Peele & Brodsky, 2000). One study of a large, mixed‐sex, multinational population reported a U‐shaped relationship between drinking level and BDI depression score: 19.3% of nondrinkers, 16.3% of moderate drinkers, and 20.0% of heavy drinkers scored above a threshold level on a curtailed BDI (O'Donnell et al., 2006). Demographics such as age may change the shape of this curve: in an older population with an average age of 62, moderate drinking was again associated with reduced risk, but high drinking did not increase risk, forming a “J‐shaped” association (Liang et al., 2021). This J‐shaped signal has reoccurred in the clinical data many times. A meta‐analysis interested in depression symptom risk and drinking level found the same decreased risk for low‐to‐moderate drinkers compared with abstinent individuals, though heavy drinking does not significantly increase risk unless heavy drinkers are compared to all nonheavy drinkers. A diagnosis of AUD, however, does increase risk for depressive symptoms (Li, Wang, et al., 2020). While this “protective” effect of moderate drinking is consistent, moderate drinking may be associated with other protective lifestyle choices (e.g., a healthy diet, increased sociability, behavioral inhibition associated with moderation in general), and, depending on the study, “nondrinker” categories may include those who are abstaining following previous psychiatric risk (Lina‐Jolien et al., 2022). On the other hand, causal evidence exists for a direct relationship between moderate drinking and lowered depression risk, according to one group (Visontay et al., 2023).

In addition, ongoing MDD may worsen abstinence efforts and treatment outcomes (Dandaba et al., 2020; Greenfield et al., 1998). In one meta‐analysis of double‐blind randomized controlled acamprosate trials, researchers found that having depression markedly affected abstinence, reducing the percent of days abstinent and the proportion of patients who maintained abstinence. Acamprosate improved depression; though when accounting for the percent of days abstinent, its effect becomes nonsignificant, implying that acamprosate improves depression indirectly via abstinence (Lejoyeux & Lehert, 2011).

In the preclinical literature, testing during the course of drinking is rare, likely because most tasks involve locomotion, meaning alcohol intoxication may affect the interpretation of the results. The foremost anhedonia task, SPT, is a consummatory choice assay utilizing drinking tubes resembling those in 2 BC, which could theoretically interfere with expectancies during the drinking period. A recent study found opposing effects of alcohol immediately following and 24 h after the fourth day of drinking‐in‐the‐dark (DID), a 2‐h binge access protocol where animals receive one tube of 20% alcohol 3 h into the dark part of their cycle. Male C57BL/6 (B6) mice showed anxiolysis in the EPM, reduced immobility in the FST, and increased sociability immediately following DID, but after 24 h of abstinence, the groups reversed direction, excepting the sociability effects (Simon et al., 2023). These results seem to suggest that acute intoxication has antidepressant‐like and anxiolytic effects, which, unsurprisingly, are some of the desired effects of alcohol intoxication in humans (Gilman et al., 2008). We cannot be certain, however, whether animals are drinking to attenuate the 24 h phenotype or whether they drink because they find alcohol positively reinforcing. Like all animal models of psychiatric disorders, depression assays give us an incomplete view of components of MDD in humans, and thus, understanding how current drinking affects mood measures has been largely unsuccessful. In humans who suffer from both disorders simultaneously (or, as will be discussed, humans who start treatment and are in withdrawal and early abstinence), it is likely that depression questionnaires and surveys are administered to patients who are not currently intoxicated, patients who may more resemble the animals who have had 24 h of abstinence. The field could benefit from affective measures taken repeatedly (where applicable) during a drinking history in animals that are not currently intoxicated; this could be achieved in animals during intermittent alcohol consumption on days when alcohol is not provided. Such measures might be translationally equivalent to affective measures taken in humans at the time of initiation of AUD treatment, providing a baseline for interpretation of how subsequent abstinence affects mood.

To summarize, ongoing AUD and MDD clearly overlap in the human literature, but preclinical studies have yet to model this sort of comorbidity during the drinking period, likely because of the locomotor confounds and anxiolysis from acute intoxication (Gilman et al., 2008; Knight et al., 2020; Simon et al., 2023). Some of the mouse literature shows mixed negative affect at early timepoints following ethanol vapor exposure specifically: anxiety in EPM in male B6 mice 4–6 h into abstinence (Kash et al., 2009), no difference in EPM in male B6 or DBA/2J mice at 5–10 h into abstinence (Finn et al., 2000), and no difference between groups in the FST in male B6 mice 8 h into abstinence (Maldonado‐Devincci et al., 2016). This vapor exposure method, known as Chronic Intermittent Ethanol (CIE) exposure, is typically carried out in cycles, whereby the alcohol group is exposed for four consecutive days for 16 h per day (Becker & Lopez, 2004); thus, inherent in this protocol is an 8‐h withdrawal period, allowing animals to metabolize ethanol completely before the next exposure (Becker, 1994). Testing for negative affect during the 8‐h period following the last CIE exposure could be thought of as “within” or “during” the drinking paradigm, though it could also be argued that animals are in acute withdrawal “after” alcohol exposure similar to patients entering treatment who present with depressive symptoms. Holding the timescale of effects of alcohol constant across species, early timepoints after drinking in animals could be equated to times between drinking bouts in humans with AUD. This discussion begs the question—is acute withdrawal between exposures “after” or “during” drinking?

### After drinking

By‐and‐large, in individuals seeking treatment for AUD, depression symptoms spike in early abstinence and dissipate with continued abstinence (Foulds et al., 2015). Clinical studies tracking these patients over continued abstinence show consistent reductions in the severity of symptoms and the percent of patients who remain depressed (Brown & Schuckit, 1988; Hallgren et al., 2018; Liappas et al., 2002; Mandić‐Gajić et al., 2015; Petit et al., 2020). See Table 2 for a summary of this literature. In all of these studies, while most participants report depression relief, some remain clinically depressed, highlighting important individual differences in affective trajectories following the cessation of drinking. While several studies have presented null results regarding the relationship between drinking level and depression risk in abstinence (Schouten et al., 2023; Sullivan et al., 2005), it appears to be relevant in an older (50+ y.o.) population (Keyes et al., 2019). Additionally, underlying drinking motives, such as using alcohol to cope, may associate with residual depression in abstinence (Kurihara et al., 2024). Thus, while on the whole, extended abstinence in humans is associated with the abatement of depression, certain state, trait, and demographic variables may predispose individuals to prolonged affective disturbance. Indeed, according to Blackford et al. at the 2024 Research Society on Alcohol annual meeting, while over 90% of patients see affective improvement with abstinence and time, around 5% of patients show persistent affective disturbance (“Speaker Abstracts,”, 2024). This recovery pattern has also occurred in other populations (Horváth et al., 2020; Rabinowitz et al., 2023).

**TABLE 2:** Clinical literature tracking depression in abstinence.

Clinical depression in abstinence		
Manuscript	*N*; demographics	Main findings
Brown and Schuckit (1988)	191; all male	42% clinically depressed at entry; 6% remained depressed 2 weeks later
Liappas et al. (2002)	28; 14.3% female	Significant reduction in HDRS after 4 weeks of abstinence
Worley et al. (2012)	237; 10% female	Longitudinal tracking of veterans in treatment. Reduction in substance use at follow‐ups tracked with reduction in HDRS
Foulds et al. (2015)	11 study meta‐analysis	All 11 studies reported improvement from baseline to the end of study. Improvement tended to occur in 3–6 weeks
Mandić‐Gajić et al. (2015)	100; all male	78 patients clinically depressed at entry; 34 patients depressed at 4 weeks; 15 patients depressed at 8 weeks; reduction of BDI and HDRS at both timepoints
Hallgren et al. (2018)	78; 19.2% female (prazosin trial follow‐up)	Regardless of prazosin treatment, negative affective symptoms and feelings decreased over a 12‐week period
Petit et al. (2020)	81; 36% female	43% presented with severe depression based on BDI scores; 18 days later, 19% remained in severe category
Horváth et al. (2020)	303; 40.59% female	Three psychopathological trajectories identified in 12‐step program: low severity with mild decrease (48.5%), moderate severity with large decrease (35.2%), high severity with moderate decrease (16.2%)
Rabinowitz et al. (2023)	6197; 43% female	Three distinct depression trajectories identified in treatment: increasing moderate depressive symptoms (14%), persistent moderate depressive symptoms (7%), and remitting mild depressive symptoms (78.99%), indicating improvement in most patients

Anhedonia, a depression symptom defined as difficulty in deriving pleasure from previously rewarding activities, associates negatively in abstinence with recovery prognosis, often better than omnibus depression scores. For example, in a study of veteran AUD patients (*n* = 95, 18.9% female), MDD diagnosis itself was not predictive of relapse, but higher treatment entry scores in the anhedonia portion of the MASQ predicted relapse (Nguyen et al., 2020). In another, state anhedonia at entry, but not trait anhedonia or overall depression score, predicted depression 18 days later in women only (Petit et al., 2020). Anhedonia was one of the more persistent symptoms in the literature in Table 2 (Brown & Schuckit, 1988; Mandić‐Gajić et al., 2015). This body of literature points toward early reward‐related dysfunction as markers of relapse and persistent depressive symptoms in abstinence. Indeed, postquit anhedonia associates with craving in substance use disorders (SUDs) (Garfield et al., 2014), and a more recent review has described anhedonia's effect on SUDs and its interaction with MDD comorbidity: anhedonia associates with SUDs and worsens treatment outcomes and prognosis, especially among those with comorbid mood disorders (Destoop et al., 2019). Early withdrawal anhedonia and drug craving are also key components of a proposed affective disorder known as postacute alcohol withdrawal syndrome (PAWS) in which this early reward‐related dysfunction is associated with protracted mood dysregulation months into abstinence (Bahji et al., 2022). Characterization of PAWS is not fully explored, though its association with anhedonia highlights the importance of this depression symptom in the human experience of alcohol abstinence.

Preclinical models of AUD‐to‐MDD comorbidity involve exposing animals to an alcohol history, removing the alcohol, and then probing for negative affect sometime during the forced abstinence period (Bloch et al., 2022). Exposure may be involuntary via inhalation of alcohol vapor or voluntary using either continuous/intermittent 2BC, DID, or operant self‐administration. Each drinking paradigm produces different variability profiles in negative affect. Groups using vapor exposure tend to test within the first week when animals are in acute withdrawal, and results here are rather modest, corroborating previous conclusions on negative affect following voluntary vs. involuntary intake in mice: voluntary intake garners more robust mood disturbances (Bloch et al., 2022; Holleran & Winder, 2017). DID drinking and intermittent 2BC produce variable results at these early time points, though there are comparatively few studies testing negative affect following intermittent 2BC (Bloch et al., 2022; Wang et al., 2021). The least variable drinking paradigm is continuous 2BC, especially 2–3 weeks following cessation of drinking, with multiple publications finding disturbed affect in FST, SPT, and NSFT during this period. Early deficits within the first 3 days of abstinence are found in EPM and LDB in the continuous 2BC model, while FST at this time point is less robust (Bloch et al., 2022; Gong et al., 2017; van Rijn et al., 2010; Vranjkovic et al., 2018). This is in line with the understanding that anxiety‐like behavior is most prevalent in early withdrawal in B6 mice rather than depression‐like phenotypes (Holleran et al., 2016), which contrasts with the clinical prevalence of both anxious and depressive symptoms in early withdrawal and abstinence.

Comparing across species, there is clear incongruence in the time lines of depression in abstinence. In humans, acute alcohol withdrawal can include depression itself (Canver et al., 2024; Heilig et al., 2010; Trevisan et al., 1998), and indeed, many patients display depressive symptoms at treatment entry (Brown & Schuckit, 1988; Lejoyeux & Lehert, 2011; Worley et al., 2012). In mice, however, affective disturbance is variable acutely across drinking paradigms, but it is most reliable 2–3 weeks following cessation of continuous 2BC access (Bloch et al., 2022). At 2–4 weeks in humans, depression symptoms are waning and tend to continue to diminish for up to 8 weeks (Brown & Schuckit, 1988; Hallgren et al., 2018; Lejoyeux & Lehert, 2011; Liappas et al., 2002; Mandić‐Gajić et al., 2015; Petit et al., 2020). This discrepancy across species calls into question what abstinence‐mediated negative affect in mice is modeling when there is a temporal mismatch between models. Additionally, the symptom clusters most associated with relapse and continued mood problems during abstinence are more related to craving and anhedonia in humans, which may be markers for dependence across patients, more so than anxiety as in the preclinical 2BC model. There are a couple of confounds that could partially explain the discrepancy.

The time lines of abstinence (e.g., acute withdrawal vs. protracted abstinence) have been considered equivalent across species, but this is likely not the case. Mice and rats metabolize alcohol faster than humans, shortening their experience with alcohol's pharmacological effects (Nelson et al., 1957); abstinence time lines may be similarly collapsed in animals. In humans, alcohol withdrawal syndrome includes “early withdrawal,” which can start 6 h after a period of lowered intake, lasting up to 48 h, followed by “late withdrawal,” which can last up to 2 weeks. Psychiatric symptoms, including mood‐related symptoms resembling depression, peak during early withdrawal and abate slowly during late withdrawal (Jesse et al., 2017). Early abstinence is less well‐defined, often overlapping with the withdrawal period and lasting 1–4 weeks after cessation—continued abstinence during this period is negatively correlated with relapse and positively correlated with the abatement of depressive symptoms (Dunn et al., 2017; Lejoyeux & Lehert, 2011). Protracted abstinence is much more long term, extending months to years after drinking ends and may include PAWS (Bahji et al., 2022). One review attempts to conciliate across species by highlighting differences in time lines by providing equivalent time periods for rodent models based on the detection of similar symptoms and neurobiological markers: acute withdrawal lasts up to 48 h and includes CNS and autonomic hyperexcitability and tremors, early abstinence lasts up to 2 weeks and includes tremors and additional affective disturbance, and protracted abstinence extends past 1 month where affective disturbance persists (Heilig et al., 2010). While this supports the idea that human and animal time lines are not equivalent, they do not match the prominent negative affect observed 2–3 weeks following cessation (Bloch et al., 2022), a time point that is neither early nor protracted according to the purported framework (Heilig et al., 2010). Additionally, this comparison neglects the negative affect seen in many patients who enter into abstinence‐based treatment (Trevisan et al., 1998). It also does not align with PAWS, which has been reported to occur in protracted abstinence months later in humans (Bahji et al., 2022); the equivalent timepoint in animals would be after 1 month of abstinence, but research in this area is scantier. Some groups observe affective disturbance following 28 days of abstinence in rats, nearing the 1‐month cutoff between early and protracted abstinence (Li et al., 2019; Valdez et al., 2002). EPM disturbances 6 and 12 weeks after CIE have also been observed (Zhao et al., 2007). In adolescent and adult B6 mice of both sexes, a more protracted view at 70 days after 14 days of DID yields few differences between history groups in FST, LDB, and MB (Jimenez Chavez et al., 2020). Thus, extending the abstinence period in rats and mice to better reflect the PAWS time line in humans still yields an unclear picture, but more research may be necessary.

Another confound in comparing clinical to preclinical studies is the spotlight of self‐selection. Most participants are treatment seekers – unlike forced abstinence imposed upon rodents at the end of the drinking period, these participants chose abstinence by entering into a treatment center and were then recruited for these clinical studies. One attempt to circumvent this is to examine MDD symptomatology in prison populations where abstinence is assumed to be more forced. One such study based in Santiago, Chile followed the course of depression in 80 inmates with significant MDD from admission to a 1‐year follow‐up; 13 of these 80 inmates had comorbid AUD. All 13 AUD+MDD participants were not significantly depressed at the follow‐up date (Baier et al., 2016). In an all‐male Greek inmate population (n = 101), depressive symptom severity was not significantly associated with prior SUDs (Kastos et al., 2022). Another study demonstrated continued improvement over 18 months of mental health endpoints especially in those subjects with alcohol abuse beforehand in a male Dutch sample (n = 1,664). The steepest incline in prognosis among inmates with SUDs was from admission to 3 months later (Dirkzwager & Nieuwbeerta, 2018). Though incarcerated populations come with their own set of biases, the situation of inmates may resemble preclinical forced abstinence more closely than treatment populations, and it seems that studies in inmates tend to show a similar decrease in depressive symptoms as abstinence continues.

## CROSS‐SPECIES ANTIDEPRESSANT EFFICACY IN REDUCING DRINKING & ASSOCIATED NEGATIVE AFFECT

Another dataset that bears on the relationship between MDD and drinking involves the study of whether antidepressants are efficacious for excessive drinking. To the extent that alcohol intake involves self‐medication for an affective disorder, logically, amelioration of the affective piece should reduce the negatively reinforcing value of alcohol consumption. Fortunately, there have been extensive studies of how antidepressants affect alcohol consumption both clinically and preclinically. Though antidepressants showed promise by reducing drinking in AUD patients in trials in the 1980s (Gorelick, 1989), they are not currently recommended for the treatment of AUD for key reasons. First, they do not consistently reduce intake. For example, daily fluoxetine, a selective serotonin reuptake inhibitor (SSRI), did not reduce drinking compared to placebo in one study (Naranjo et al., 1990) and since then other SSRIs have failed in placebo‐controlled studies (Torrens et al., 2005). In many cases, SSRIs can increase alcohol consumption (Charney et al., 2015; Pettinati et al., 2000; Wood & Rehm, 2024) or serve as a risk factor for AUD itself (Brookwell et al., 2014). Second, common antidepressants interact with alcohol, especially tricyclics and MAO inhibitors (Weathermon & Crabb, 1999). SSRIs have markedly less interactions with alcohol, but it is not recommended to mix them. Though based entirely on case reports, a behavioral interaction referred to as a “syndrome of pathological alcohol intoxication,” characterized by a leftward shift in perceived intoxication, increased disinhibition, and memory impairment has been described (Menkes & Herxheimer, 2014).

In preclinical studies, antidepressants typically reduce drinking. Tricyclic antidepressants (TCAs) blocking serotonin and/or norepinephrine reuptake have been shown to reduce free‐choice EtOH drinking in male Long Evans rats (Daoust et al., 1984) and reduce operant self‐administration in CIE‐exposed male Wistar rats along with the SNRI milnacipran (Simon O'Brien et al., 2011). SSRIs have also been efficacious, reducing 2BC drinking in male B6 mice (Ho et al., 2016) and operant self‐administration in dependent male Wistar rats (Simon O'Brien et al., 2011). However, unlike in humans, these drugs often show acute effects even after single dosing, calling into question their translatability as most classes of antidepressants require repeated dosing over months and weeks to show any behavioral or psychological improvements.

Antidepressants can be prescribed off‐label to human patients with comorbid AUD and MDD, though research here is mixed, often not affecting comorbidity models (Lejoyeux & Lehert, 2011). SSRIs seem to be less effective than TCAs in the comorbid patients for reducing alcohol use during abstinence, though TCAs have much more dangerous interactions with alcohol (Torrens et al., 2005; Weathermon & Crabb, 1999). One meta‐analysis of 33 studies and over 2000 participants concluded that there is only “low‐quality evidence” after adjusting for biases that antidepressants have a positive effect on both disorders, though adverse effects of SSRIs specifically are minimal, and they may be ineffective‐to‐modestly helpful for AUD + MDD patients (Agabio et al., 2018). Overall, antidepressants continue to be an off‐label treatment for those with comorbid AUD + MDD, but research is mixed, and continued investigation is needed (Fischler et al., 2022). Preclinically, antidepressants have been successful in reducing negative affect in abstinence in mice (Fraga‐Junior et al., 2022; Li, Lu, et al., 2020; Stevenson et al., 2009) and rats (Campos‐Cardoso et al., 2021; Uzbay, 2008), which is in contrast with clinical data. Honing the translatability of preclinical models of this comorbidity could aid in the discovery of more effective pharmacotherapies for patients, whether that be with lines and strains that, behaviorally, more closely resemble these patients during abstinence, or with alternative, rapid antidepressants.

Psychedelic compounds like psilocybin and the dissociative ketamine may be used in human MDD treatment (Dawood Hristova & Pérez‐Jover, 2023; Yavi et al., 2022), and the administration of psilocybin (Bogenschutz et al., 2022) or ketamine (Dakwar et al., 2020) in combination with psychotherapy has shown success in reducing drinking in patients with AUD. In mice, ketamine injection reduces signs of abstinence‐related negative affect when given at the onset of abstinence (Holleran et al., 2016; Vranjkovic et al., 2018) and attenuates depressive‐like behavior in rats in the absence of ethanol (Crisanti et al., 2020) as does psilocybin in mice and rats (Hesselgrave et al., 2021; Shore et al., 2024). Ketamine has also reduced intake in some strains of mice (Crowley et al., 2019) though not in mice selectively bred to drink alcohol (Ardinger et al., 2021). Single‐dose psilocybin has been shown to reduce EtOH consumption in male but not female B6 mice (Alper et al., 2022) and reduces alcohol‐seeking in alcohol‐dependent rats (Meinhardt et al., 2021), though it may have dose‐dependent effects on locomotion that should be mentioned as a possible confound explaining this attenuation (Pedicini & Cordner, 2023) Furthermore, animals often respond to single doses of classic antidepressants like SSRIs (Petit‐Demouliere et al., 2005) while they take weeks to be effective in humans. In most studies utilizing ketamine or psychedelics, one dose is enough to show improvement of AUD or MDD symptoms in both rodents and humans.

## NEGATIVE AFFECT ACROSS STRAINS/LINES

Among high‐drinking selected mice lines such as the High Alcohol‐Preferring lines (HAP, cHAP, and cHAPxHDID), only the crossed High Alcohol‐Preferring (cHAP) mice have been tested for depressive‐like behavior following an alcohol history. This line is a cross between the HAP1 and HAP2 lines (Oberlin et al., 2011) and exceeds B6 levels of home cage drinking, readily preferring alcohol to water and accruing intoxicating BECs (>200 mg/dL) in 2BC (Matson et al., 2013). After 5 weeks of 2BC and either 1 or 3 weeks of forced abstinence, male and female cHAP mice show no significant increases in NSF latency and FST immobility; in the SPT, however, alcohol‐drinking animals increase sucrose consumption and preference following 1 week of abstinence (Winkler & Grahame, 2024). Additionally, an anxiety‐like phenotype in female cHAPs in the EPM and OFT following 7 months of voluntary drinking was accompanied by thiamine deficiency, brain damage, and neuroinflammation (Xu et al., 2021). Thus, a relatively longer drinking period may engender negative affect for cHAPs. We would note that the near lack of a withdrawal depression phenotype in cHAP mice, notwithstanding their robust voluntary alcohol intake, is consistent with a broader literature on the relationship between voluntary alcohol drinking and withdrawal susceptibility: the higher the voluntary alcohol intake, the lower the level of handling‐induced convulsions following exposure to chronic alcohol vapor (Metten et al., 1998). Notably, this includes High Alcohol‐Preferring mice, which show almost no handling‐induced convulsions following repeated rounds of alcohol vapor exposure that are normally effective in eliciting withdrawal in inbred mice (Lopez et al., 2011). It is not clear whether withdrawal‐related affect would be correlated with withdrawal‐related convulsive activity. But to the extent that both seizures and mood changes following drinking represent homeostatic disturbances, one can argue that the lack of depression in high‐drinking populations is consistent with their lack of physical withdrawal following ethanol exposure.

Mixed findings occur in high‐drinking rat lines, which generally do not achieve the BECs observed in mice during 2BC drinking. Marchigian Sardinian alcohol‐preferring rats (msP rats) exhibit decreased immobility in the FST in both sexes and reduced anxiety in males following alcohol consumption and forced abstinence (Ciccocioppo et al., 2006). Fawn‐Hooded (FH/Wjd) rats are one unique model of comorbidity that independently show elevated EtOH preference (5–6 g/kg/day) along with alcohol‐naïve negative affect compared to selectively bred, alcohol‐preferring P rats (Overstreet et al., 2007). They also have exhibited increased anxiety‐like behavior in the EPM following 4 weeks of 2BC and 24 h of abstinence (Gong et al., 2017). In comparison with Wistar rats, FH/Wjd rats also show increased plasma levels of corticosterone following the FST, suggesting higher stress reactivity (Knapp et al., 2018). These animals, however, are decidedly underutilized in preclinical comorbidity studies.

## FUTURE DIRECTIONS

AUD + MDD models for concurrent comorbidity have been tenuous in animal models. There are many confounds (e.g., ataxia and consummatory behavior) to subjecting animals to behavioral tests under the influence of alcohol intoxication. Human AUD patients are driven to maintain alcohol consumption through a combination of positive and negative reinforcement, implying that there are lapses in drinking whereby mood‐related symptoms are experienced (Cho et al., 2019). Cycles of withdrawal are often thought to be a part of the AUD diagnosis, indicating that a patient is physically dependent on alcohol (American Psychiatric Association, 2013). Therefore, it would be helpful in the preclinical alcohol field to define this early withdrawal period lasting up to 48 h postcessation (Heilig et al., 2010) as contained within the AUD model, whereby negative affect measured during this time could be thought of as MDD‐like symptoms accompanying withdrawal between bouts of drinking.

This reframing of AUD + MDD comorbidity as appearing in early withdrawal aligns more with the human experience of negative affect during this period (Canver et al., 2024) and allows for prospective study in animals of another pathway of comorbidity, that is, when drinking precedes MDD. Abstinence‐related affective disturbance studies tend to run batteries of behavioral tests at a single time point, likely because many of these tests can only be run at once due to the possibility of habituation to the apparatus. By probing animals at the onset of abstinence using one or two assays, then another different assay during the “protracted” period, researchers can obtain multiple readouts on affective behavior in a longitudinal fashion, allowing for paired correlational analyses rather than only the binary “yes/no” manifestation of negative affect. In humans, there is evidence that anhedonia in early withdrawal is correlated with relapse and continued affective problems in early and protracted abstinence (Destoop et al., 2019; Garfield et al., 2014). Thus, as a test of the translatability of these findings, anhedonia could be measured at T1 with FST/NSFT/TST/etc. measured at T2. The intervening period between these time points should reflect the time between early withdrawal and protracted abstinence, possibly a month or longer in animal models (Heilig et al., 2010).

Anhedonia is primarily measured using SPT in animals, where decreased sucrose preference compared to water controls is seen as evidence for anhedonia (Liu et al., 2018). While this assay is fast and simple, as it is essentially a 2BC variant, its resemblance to 2BC may be a confound as alcohol history animals have choice experience compared to a control group with two water bottles. One approach for redefining anhedonia tasks is to break up anhedonia into consummatory and motivational anhedonia, where the former reflects the reduction in “liking” a reward and the latter is akin to a reduction in “wanting” a reward (Gencturk & Unal, 2024). In addition to measures of total quantity, consummatory anhedonia could be refined by measuring microstructure of consumption from a single solution of sucrose, similar to frontloading behavior during DID (Ardinger et al., 2022), in which greater frontloading indicates greater avidity. Motivational anhedonia could be measured using a progressive ratio operant task (Gencturk & Unal, 2024). In humans, anhedonic responses are often measured to “secondary reinforcing stimuli” such as money or pleasant imagery, while the SPT relies on a primary reinforcer (Markov, 2022). Motivational anhedonia tasks could place the primary reinforcer behind a secondary one, thus providing face validity to the clinical picture. Moving forward, it is important to determine which piece of anhedonia associates with more protracted negative affect.

Finally, there is likely not just one pathway to AUD. The prevalence of MDD among people with AUD is somewhere between 28 % and 33%, meaning that MDD is not guaranteed even in the presence of AUD (McHugh & Weiss, 2019; Petrakis et al., 2002). Those with comorbid AUD + MDD may reflect a “relief drinking” pattern of intake rather than a “reward drinking”‐based pattern (Verheul et al., 1999), an idea evolved from the “Type 1” vs. “Type 2” alcoholism model (Cloninger et al., 1988). A relief drinker skews toward drinking for alcohol's anxiolytic effects, driven by negative reinforcement, while a reward drinker is motivated by the rewarding effects of intoxication, that is, positive reinforcement. So while AUD and MDD overlap, they may do so more strongly in a population of relief drinkers. This could explain sex differences in comorbidity (Goldstein et al., 2012), which may relate to reports of increased negative reinforcement drinking in women (Peltier et al., 2019). The alcohol field could apply the individual differences preclinically in drinking motives to strains and sexes, acknowledging that some strains may be more likely to engage in relief (e.g., B6) versus reward (e.g., HAP mouse lines) drinking. As noted in the introduction, current preclinical models of relief drinking, drinking via negative reinforcement, or MDD‐to‐AUD comorbidity involve applying chronic or acute stress followed by access to alcohol to detect increases in drinking. In the absence of stress, the propensity of an inbred strain or selected line of rats or mice to exhibit behavioral correlates of depressive‐like behavior may or may not correlate with that line or strain's capacity to drink alcohol. For example, inbred strains differ in FST immobility and sucrose preference when compared in the same study, with male and female B6 mice exhibiting increased FST immobility compared to DBA/2 and FVB/N strains and DBA/2 mice exhibiting lower sucrose preference compared to the other two strains (Eltokhi et al., 2021). These strains consume alcohol to different extents in DID and 2BC, with the general pattern being B6 > FVB/N > DBA/2 for both intake and BECs in both paradigms (Rhodes et al., 2007). This sort of independent phenotyping could be applied to high‐drinking selected lines as well in order to detect drinking–depression correlations across behavioral categories including anhedonia, helplessness, and despair.

While sex differences are not the focus of this review, we agree that they should be a focus in this subfield of alcohol research, especially in light of the shrinking gap in alcohol‐related outcomes between the sexes in humans (McCaul et al., 2019) and the continued sex disparity in depression rates whereby women are disproportionately affected (Salk et al., 2017). Many preclinical behavioral tasks were designed with male rodent behavior in mind, and a refocusing of what negative affect looks like in female mice and rats is overdue. A recent review more comprehensively evaluates the sex differences in negative affect following a history of alcohol drinking, and the authors find variable results across categories of affective behavior depending on species, strain, drinking paradigm, and abstinence time (Salazar & Centanni, 2024).

## CONCLUSION

**FIGURE 1:**
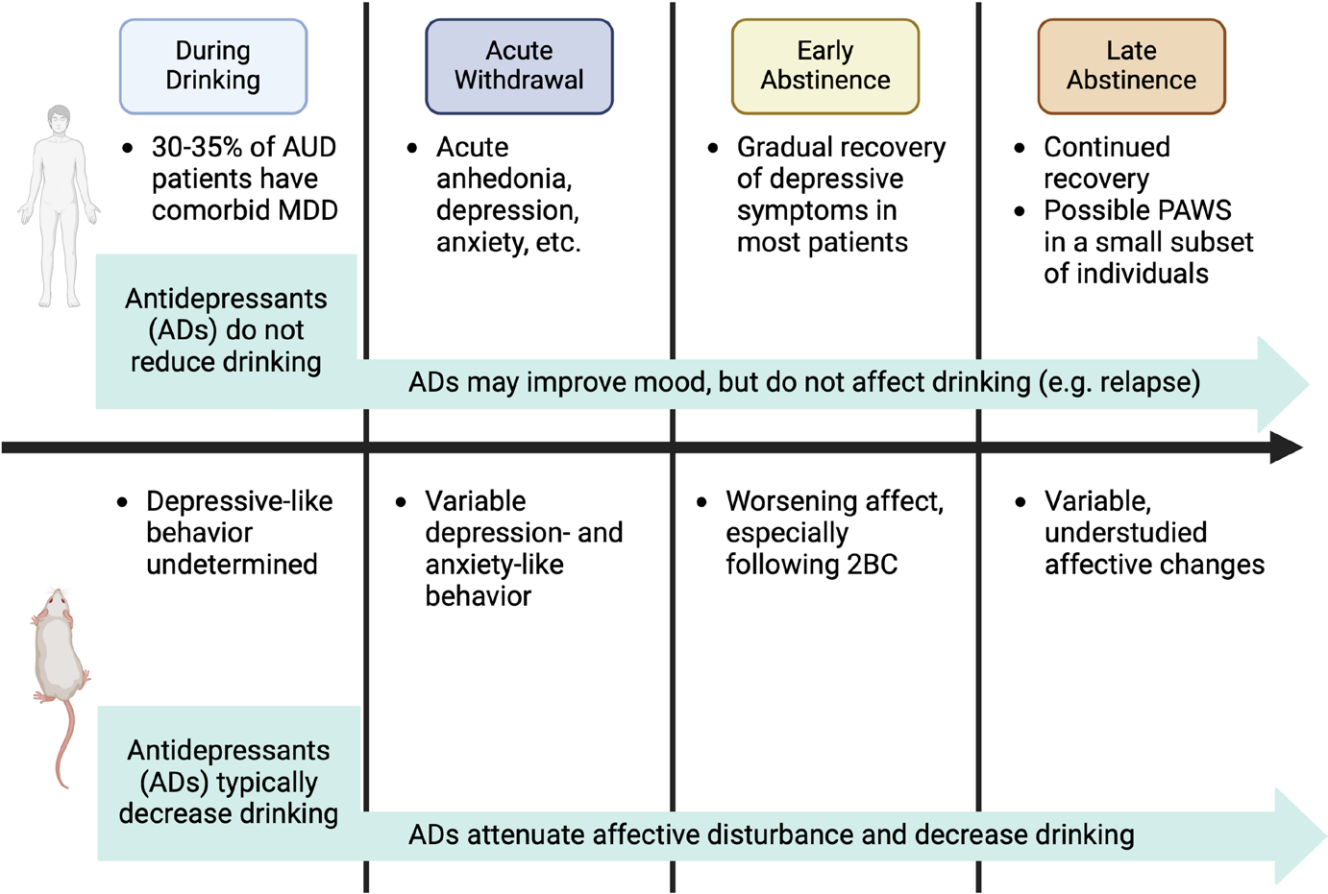
Summary of this review with focus on species time line divergence and antidepressant efficacy. Clinically and preclinically, acute withdrawal can last up to 48 h. In humans, early abstinence can last up to 4 weeks; in animals this period lasts up to 2 weeks, though most affective disturbance is found at the 2–3 week mark. Late, or protracted, abstinence begins 3+ months after cessation in humans and 1+ month in animals.

This review compares preclinical models and clinical research of AUD + MDD comorbidity, including differences across species in models and time lines of concurrent AUD + MDD and MDD that follows AUD; species disparities in the capacity of common antidepressants to reduce drinking and abstinence‐related affective disturbances; and high‐drinking rodent lines' affective responses in abstinence. See Figure 1 for a summary. We also discussed suggestions and future directions including a reframing of the modeling of concurrent and subsequent MDD comorbidity; a focus on early anhedonia's correlation to protracted affective states; novel models of preclinical anhedonia; and the incorporation of reward and relief drinking types into the preclinical space, reflecting the breadth of individual differences within and between human populations.

## CONFLICT OF INTEREST STATEMENT

The authors declare no competing interests.

## Data Availability

Data sharing not applicable to this article as no datasets were generated or analysed during the current study.
